# Can machine learning predict non-suicidal self-injury? A systematic review and meta-analysis

**DOI:** 10.3389/fpubh.2026.1763121

**Published:** 2026-04-30

**Authors:** Qianhui Wen, Rong Luo, Qian Wang

**Affiliations:** 1 Department of Pediatrics, West China Second University Hospital, Sichuan University, Chengdu, Sichuan, China; 2 Key Laboratory of Birth Defects and Related Diseases of Women and Children (Sichuan University), Ministry of Education, Chengdu, Sichuan, China

**Keywords:** deep learning, machine learning, meta-analysis, non-suicidal self-injury, systematic review

## Abstract

**Background:**

Non-suicidal self-injury (NSSI) is common among adolescents and young adults and remains difficult to detect early using conventional approaches. machine learning (ML) has increasingly been applied to develop prediction models for NSSI.

**Methods:**

We conducted a systematic review and meta-analysis of studies that developed ML models for NSSI prediction, as defined by the original study authors. Multiple databases were searched from inception to June 28, 2025. Model performance, including the area under the curve (AUC), sensitivity, and specificity, was synthesized using a bivariate random-effects model. Risk of bias was assessed using PROBAST+AI.

**Results:**

Twelve studies involving 33,366 participants were included. In the primary model-level analysis, ensemble models showed relatively favorable pooled discrimination, with a pooled AUC of 0.83 (95% CI: 0.79–0.86), sensitivity of 0.78 (95% CI: 0.68–0.85), and specificity of 0.73 (95% CI: 0.58–0.84). Single models showed lower performance (AUC: 0.68, 95% CI: 0.64–0.72). Only one study evaluated a deep learning (DL) model (AUC = 0.70), and this estimate should therefore be interpreted cautiously. Across all 19 models, the pooled AUC was 0.75 (95% CI: 0.71–0.79). Substantial heterogeneity was observed, and the apparent advantage of ensemble models was not sustained in the study-level sensitivity analysis. Most studies were judged to be at high risk of bias in the analysis domain.

**Conclusions:**

ML models show promise for identifying NSSI-related risk, but current evidence supporting true prospective prediction remains limited. The evidence base is constrained by substantial heterogeneity, a high risk of bias, and the predominance of cross-sectional studies. Prospective multicenter studies with external validation and standardized reporting are needed before ML-based models can be translated into clinical or public health practice.

**Systematic Review Registration:**

https://www.crd.york.ac.uk/PROSPERO/view/CRD420251075613, identifier: CRD420251075613.

## 1 Introduction

Non-suicidal self-injury (NSSI) refers to the deliberate, repetitive destruction of one's own body tissue in the absence of suicidal intent (1). It is highly prevalent among adolescents and young adults, with an estimated lifetime prevalence of 17%−18% among adolescents worldwide (2). NSSI commonly co-occurs with mental disorders such as depression, anxiety, and borderline personality disorder (1), and it is also an independent predictor of subsequent suicidal behavior (3, 4). Given its high prevalence and clinical significance, NSSI has become a major global public health concern, underscoring the need for efficient and accurate tools for risk identification and assessment in both clinical and public health settings.

Despite this urgent need, the early identification and risk assessment of NSSI remain methodologically challenging in clinical practice. Traditional approaches rely primarily on clinical interviews and self-report questionnaires, both of which are vulnerable to recall bias, social desirability bias, and assessor bias (5). In addition, these methods provide limited timeliness and are poorly suited to dynamic risk prediction (6, 7). NSSI has a complex etiology shaped by interacting genetic, neurobiological, psychological, and socio-environmental factors (8). Conventional statistical models, constrained by linear assumptions and a limited capacity to handle high-dimensional data, may therefore be insufficient to capture the heterogeneous pathways underlying NSSI.

In recent years, machine learning (ML) has emerged as a promising approach in mental health research (9, 10). Compared with traditional statistical methods, ML is better able to handle high-dimensional and multimodal data and to capture complex nonlinear relationships and interactions among variables, thereby potentially improving predictive accuracy and generalizability (11–13). Algorithms such as support vector machines (SVMs), random forests (RFs), and neural networks (NNs) are particularly well suited to integrating heterogeneous data sources, including clinical, behavioral, neuroimaging, and electronic health record data, for the development of predictive models (14, 15). ML has already shown value in predicting a range of mental health outcomes, including depression, anxiety, and suicide risk (16–18).

However, the application of ML to NSSI prediction remains limited by substantial methodological heterogeneity and a lack of standardized reporting. Studies vary considerably in sample sources, predictor selection, data-processing pipelines, and validation strategies. Moreover, the clinical generalizability of these models is restricted because most studies have relied solely on internal validation, with little external validation in independent samples or multicenter settings (19). In addition, key measures relevant to clinical translation, such as calibration and clinical utility, are often inadequately reported. Although previous reviews have examined ML applications for broader suicide-related behaviors (20–22), meta-analyses specifically evaluating predictive performance for NSSI remain scarce. Importantly, the comparative performance of different classes of ML models in this context has not been systematically examined.

This study aimed to conduct a systematic review and meta-analysis to evaluate the discriminative performance of ML models for identifying or predicting NSSI while considering the methodological characteristics of the current evidence base. Specifically, we sought to synthesize key performance metrics, including pooled area under the curve (AUC), sensitivity, and specificity, across available studies, compare the performance of different model classes, and explore potential sources of heterogeneity. The findings may help inform the optimization, validation, and eventual translation of high-performing NSSI prediction models.

## 2 Methods

This systematic review was registered in PROSPERO (CRD420251075613) and conducted in accordance with the PRISMA statement (Supplementary Table S1).

### 2.1 Inclusion criteria

Studies were eligible if they: (1) focused on the prediction of NSSI; (2) described the development of a complete ML model; and (3) reported predictive performance metrics, such as the receiver operating characteristic (ROC) curve, C-index, sensitivity, specificity, or a confusion matrix.

### 2.2 Exclusion criteria

Studies were excluded if they were: (1) reviews, conference abstracts, brief reports, or other non-peer-reviewed publications; (2) focused solely on suicidal behaviors without a separate analysis of NSSI; (3) limited to the mechanisms, treatment, or descriptive epidemiology of NSSI; or (4) published in languages other than English.

### 2.3 Data sources and search strategy

A comprehensive search was conducted in PubMed, Web of Science, Cochrane Library, Embase, PsycINFO, and IEEE Xplore from database inception to June 28, 2025. The search strategy combined controlled vocabulary and free-text terms related to NSSI and ML. No restrictions were applied with respect to geographic region or publication period. The full search strategy is provided in Supplementary Table S2.

### 2.4 Study selection and data extraction

Study selection and data extraction were performed independently by two reviewers (QWe and QWa). Records were imported into EndNote for deduplication and screened by title, abstract, and full text. The reference lists of included studies were also manually searched to identify additional eligible articles. Using a standardized form, we extracted data on study characteristics, population characteristics, outcome definitions, model characteristics, validation strategies, and model performance measures. Specifically, extracted items included study design, sample size, data source, participant characteristics, predictor modality, model type, validation method, the reported classification threshold or operating point, and performance metrics such as AUC, sensitivity, specificity, and C-index. When a study reported multiple eligible models, data were extracted for all models that met the inclusion criteria.

Because most studies did not report 2 × 2 contingency tables for the validation set, true positives, false positives, true negatives, and false negatives were reconstructed whenever possible from the sample size, outcome distribution, and the reported validation sensitivity and specificity. When only ROC-derived performance characteristics were available, sensitivity and specificity at the reported primary operating point were used for reconstruction. The reconstructed data were independently verified by the two reviewers. Disagreements were resolved through discussion or consultation with a third reviewer (RL).

### 2.5 Threshold handling

Classification thresholds were based on the original study reports. For each eligible model, sensitivity and specificity were extracted at the reported threshold. If a validation-set cutoff was explicitly provided, that cutoff was used. Otherwise, when ROC-based characteristics were available, sensitivity and specificity at the primary operating point (e.g., the optimal Youden index) were extracted.

### 2.6 Risk of bias assessment

We used the updated PROBAST+AI (Prediction model Risk Of Bias Assessment Tool + Artificial Intelligence) to assess the risk of bias and applicability of the included studies (23). This tool is designed for prediction models developed using artificial intelligence methods, including ML, and comprises two components: an assessment of model-development quality (16 items) and an assessment of risk of bias in model validation (18 items). Both components cover four core domains: participants and data sources, predictors, outcomes, and analysis. Each item is rated as “Yes/Probably Yes,” “No/Probably No,” or “No information.” A domain is judged to be at high risk if any item is rated “No/Probably No” and at low risk if all items are rated “Yes/Probably Yes.” A study is considered to be at overall high risk if any domain in either the development or validation stage is rated as high risk. The first three domains also include an applicability assessment evaluating relevance to the intended use scenario. Two reviewers (QWe and QWa) independently assessed all included studies, with disagreements resolved through discussion and, when necessary, arbitration by a third reviewer (RL).

### 2.7 Statistical analysis

Statistical analyses were performed using R (version 4.3.2) and Stata 16.0. Sensitivity and specificity for ML-based NSSI prediction models were pooled using a bivariate random-effects model, and forest plots were generated to display the pooled estimates. Summary receiver operating characteristic (SROC) curves were constructed to visualize overall model performance, and pooled AUC values were derived from the SROC curves. Statistical heterogeneity was assessed using the *I*^2^ statistic.

In the primary meta-analysis, the analytic unit was the individual ML model evaluated on the validation set. When a study reported multiple eligible models, these models were initially treated as separate observations. Because models from the same study may share the same underlying sample and are therefore not fully independent, we also performed a study-level sensitivity analysis in which one representative model per study was retained. For studies with multiple eligible models, the primary or final model identified by the original authors was retained; if no such model was specified, the model with the most complete validation data was selected. The same bivariate meta-analytic framework, including pooled sensitivity, pooled specificity, SROC analysis, and AUC estimation, was then applied at the study level.

Potential publication bias was explored using Deeks' funnel plot asymmetry test, with *P* < 0.05 considered statistically significant. Because DL, single model, and ensemble model approaches differ substantially in structure and assumptions, subgroup analyses by model type were performed to explore heterogeneity.

## 3 Results

### 3.1 Study selection

**Figure 1:**
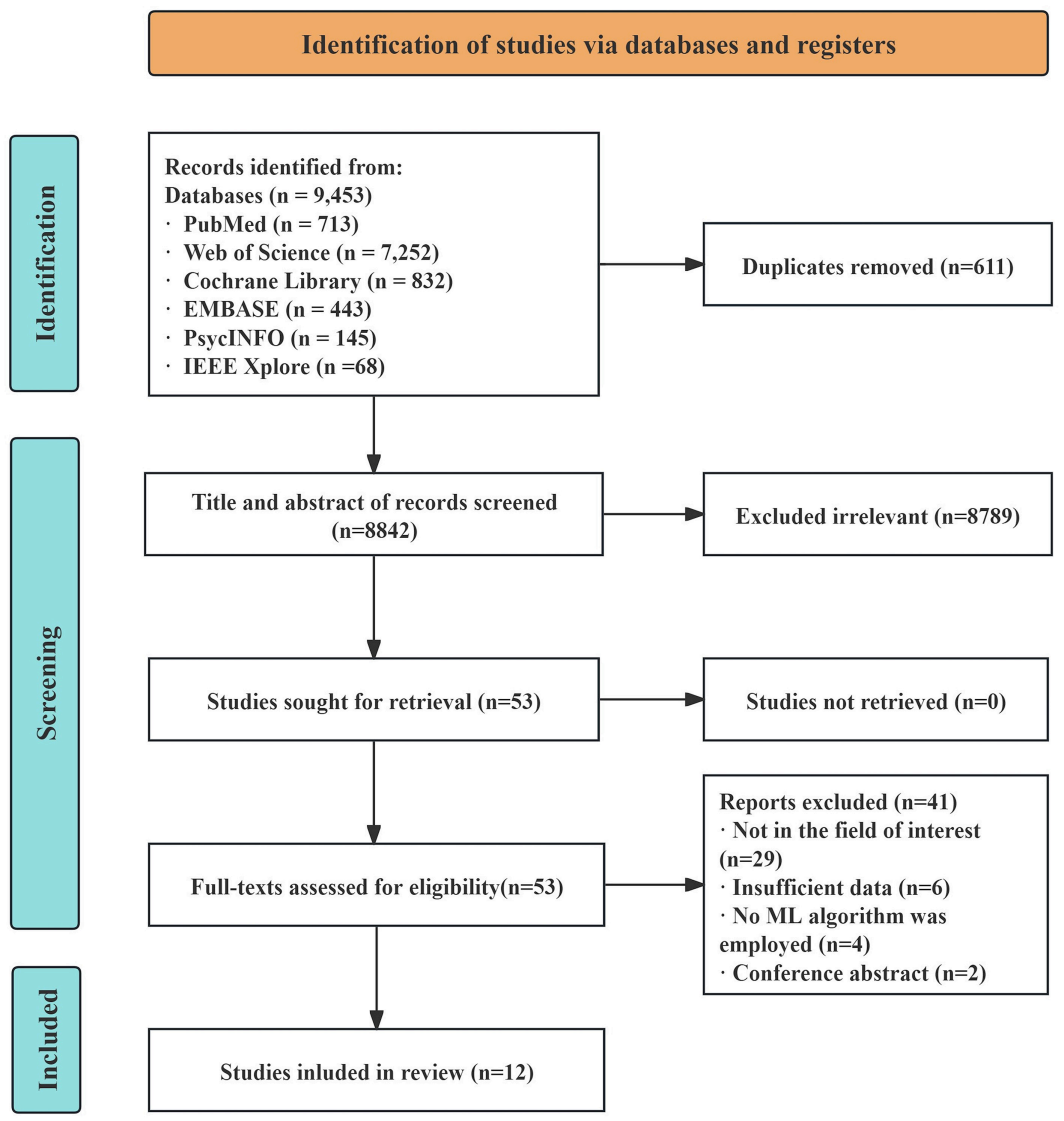
Flowchart summarizing the study selection process.

The initial search identified 9,453 records. After automatic deduplication in EndNote and manual verification, 611 duplicate records were removed. The remaining 8,842 records underwent title and abstract screening, during which 8,789 were excluded because they were irrelevant to the research question or represented reviews, guidelines, case reports, comments, letters, or other ineligible publication types. Full-text review was performed for the remaining potentially eligible articles. After detailed assessment, 41 studies were excluded for the following reasons: 29 used controversial definitions of NSSI outcomes, six lacked key outcome measures, four did not use ML models, and two were unpublished conference abstracts. Ultimately, 12 studies (24–35) met the predefined inclusion criteria. The study selection process is shown in Figure 1.

### 3.2 Study characteristics

Table 1 summarizes the characteristics of the 12 included studies, published between 2019 and 2025. Together, these studies enrolled 33,366 participants, with sample sizes ranging from 114 to 13,304. Although age-group definitions varied, all studies covered key developmental stages from adolescence to early adulthood. Geographically, most studies were conducted in China (10 studies) (24–26, 28–34), with one study each from the United States (35) and South Korea (27). Methodologically, 2 studies were multicenter (31, 32) and 10 were single center (24–30, 33–35). Study designs included 1 prospective study (35), 1 retrospective study (33), and 10 cross-sectional studies (24–32, 34). Across the 12 studies, 19 models were reported: 10 single models (25, 27, 31, 32, 34), eight ensemble models (26, 28–30, 33, 35), and 1 DL model (24).

**Table 1:** Characteristics of included studies.

Study	Country	Design/ setting	Population	Total SampleSize	Total number of cases	Prediction target	Data modality	Model type	Modelclassification	Validation strategy	Resampling/class balance	Outcomedefinition
Liang (24)	China	Cross-sectional; single-center	Adolescents with DSM-5 major depressive disorder, 13-18 y	114	77	NSSI classification	EEG	NSSI-Net (CNN-BiGRU-GAN; semi-supervised deep learning)	DL model	Cross-subject semi-supervised 10-fold cross-validation; no independent test set	Undersampling for sex-balanced subsets	DSM-5 + MINI interview
Liu (25)	China	Cross-sectional; single-center	Hospitalized adolescents/young adults with mental disorders, 15-29 y	658	335	NSSI risk prediction	Clinical	LR	Single model	Random split-sample internal validation (70/30)	None	DSM-5
Sun (26)	China	Cross-sectional; single-center	Adolescents/young adults with DSM-5 MDD, 13-25 y	488	161	NSSI classification	Clinical	XGBoost, RF	Ensemble model	Random split-sample internal validation (75/25)	None	K-SADS-PL interview
Kim (27)	Korea	Cross-sectional; single-center	Adult psychiatric patients > = 19 y	224	78	NSSI classification	Clinical	Decision tree	Single model	Apparent model only; no train-test split, cross-validation, or external validation	None	single-item clinical interview question
Chen (28)	China	Cross-sectional; single-center	Junior/senior high school students, 11–22 y	4,487	1,145	NSSI classification	Clinical	Stacking	Ensemble model	Random split-sample internal validation (70/30)	SMOTE oversampling	NSSIQ questionnaire
Bao (29)	China	Cross-sectional; single-center	Middle school students, 11–15 y	2,385	401	NSSI classification	Clinical	RF	Ensemble model	Random split-sample internal validation (70/30)	class-weight balancing	single self-report NSSI item
Jiang (30)	China	Cross-sectional; single-center	Rural junior high school students, mean age 13.68 y	2,090	800	NSSI classification	Clinical	RF, Decision Tree, SVM-Linear, SVM-Radial, SVM-Polynomial, SVM-Sigmoid	Ensemble and single models	Train/validation/test split (70/15/15)	None	Adolescents self-harm scale
Zhong (31)	China	Cross-sectional; multi-center	Adolescents from 50 schools, 10–19 y	13,304	617	NSSI risk prediction	Clinical	LR	Single model	Random split-sample internal validation (70/30)	None	Ottawa self-injury Inventory (Chinese)
Zhou (32)	China	Cross-sectional; multi-center	Students and their primary caregivers	7,967	201	NSSI risk prediction	Clinical	LR, RF	Ensemble and Single models	Stratified random split-sample internal validation (75/25)	None	Past-12-month self-report NSSI item
Kang (33)	China	Retrospective; single-center	Adults with MDD	346	77	NSSI classification	Imaging	RF	Ensemble model	10-fold cross-validation; no independent test set	None	K-SADS-PL-based interview
Yang (34)	China	Cross-sectional; single-center	Adolescents/young adults, 10–24 y	282	137	NSSI classification	Clinical	SVM	Single model	10-fold cross-validation; no independent test set	Bootstrap resampling	DSM-5
Fox (35)	United States	Prospective; single-center	High-risk self-injurious and/or suicidal adults recruited online, mean age 26.5 y	1,021	382	NSSI risk prediction	Clinical	RF	Ensemble model	Bootstrap internal validation (100 replicates); no conventional train-test split	None	Modified SITBI interview

BiGRU, bidirectional gated recurrent unit; CNN, convolutional neural network; DL, deep learning; DSM-5, diagnostic and statistical manual of mental disorders, fifth edition; EEG, electroencephalography; GAN, generative adversarial network; K-SADS-PL, schedule for affective disorders and schizophrenia for school-age children-present and lifetime version; LR, logistic regression; MDD, major depressive disorder; MINI, mini-international neuropsychiatric interview; ML, machine learning; NSSI, non-suicidal self-injury; NSSIQ, Non-Suicidal Self-Injury Questionnaire; RF, random forest; SITBI, self-injurious thoughts and behaviors interview; SMOTE, synthetic minority oversampling technique; SVM, support vector machine; XGBoost, extreme gradient boosting.

Single models mainly included logistic regression (LR), SVMs, and decision trees (DTs), whereas ensemble models primarily comprised extreme gradient boosting (XGBoost), RFs, and stacking approaches. The sole DL model used a convolutional neural network (CNN) + bidirectional gated recurrent unit (BiGRU) + generative adversarial network (GAN) architecture. All studies used internal validation; three used 10-folds cross-validation (24, 33, 34), and one used bootstrap validation (35). In terms of data type, one study used electroencephalography (EEG) data (24), one used imaging data (33), and the remaining studies were based on clinical characteristics. Outcome ascertainment for NSSI followed the definitions reported in the original studies and was based on self-report measures, clinical assessment, or record-based information, with varying reference periods and criteria.

### 3.3 Quality of the studies

**Figure 2:**
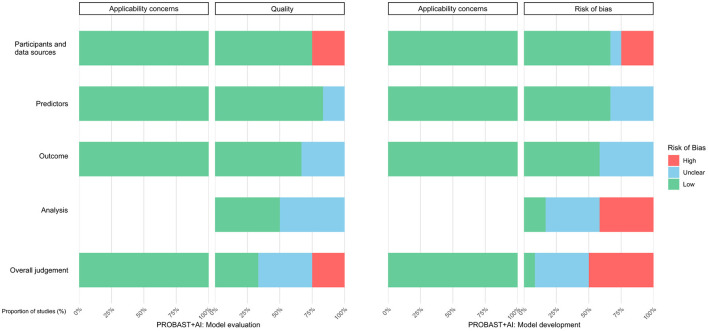
Summary of risk of bias and applicability concerns.

The risk of bias and applicability of the included studies are summarized in Figure 2.

#### 3.3.1 Domain of participants and data sources

Most studies were judged to have a low risk of bias in the participants and data-sources domain. However, Bao et al. (29), Kang et al. (33), and Yang et al. (34) were rated as having a high risk of bias because of concerns related to data sources or study design, primarily involving limited representativeness or inadequate study design.

#### 3.3.2 Domain of predictors

In the predictors domain, most studies performed well with respect to the definition, assessment, and handling of predictors, with generally consistent definitions and standardized assessment procedures. Most studies also ensured that predictor assessment was blinded to outcome information. However, Sun et al. (26) and Kang et al. (33) were judged to be at high risk of bias because of inconsistent predictor definition or assessment.

#### 3.3.3 Domain of outcomes

Overall, the outcomes domain showed a generally low risk of bias. Most studies used reasonably consistent outcome definitions and assessment methods, with an appropriate interval between predictor measurement and outcome assessment. However, Liu et al. (25), Sun et al. (26), Bao et al. (29), and Kang et al. (33) were judged to be at high or unclear risk of bias because of unclear outcome-assessment methods or missing outcome information.

#### 3.3.4 Domain of analysis

Risk of bias was notably high in the analysis domain. Sun et al. (26), Kim et al. (27), Zhong et al. (31), Zhou et al. (32), Kang et al. (33), and Yang et al. (34) were judged to be at high risk of bias because of issues such as insufficient sample size, unclear handling of missing data, overfitting, or inappropriate validation methods. In particular, several studies did not provide adequate sample-size justification or implement appropriate validation strategies during model development.

#### 3.3.5 Applicability assessment

All included studies showed good applicability. Applicability concerns across the three domains—participants, predictors, and outcomes—were judged to be low, indicating that the included models were broadly relevant to the intended use scenarios. Overall, applicability was considered favorable, with no major mismatch identified. Detailed results are presented in Supplementary Tables S3 and S4.

### 3.4 Meta-analysis

The meta-analysis results are summarized in Table 2. Among the 10 single models, the pooled AUC was 0.68 (95% CI: 0.64–0.72), with a pooled sensitivity of 0.61 (95% CI: 0.43–0.77) and a pooled specificity of 0.66 (95% CI: 0.47–0.81). Among the eight ensemble models, the pooled AUC was 0.83 (95% CI: 0.79–0.86), with a pooled sensitivity of 0.78 (95% CI: 0.68–0.85) and a pooled specificity of 0.73 (95% CI: 0.58–0.84).

**Table 2:** Predictive performance of ML models for NSSI in the primary model-level meta-analysis, by model type.

AI algorithms	*n*	Sensitivity (95% CI)	Specificity (95% CI)	AUC (95% CI)
Single models	10	0.61 (0.43; 0.77)	0.66 (0.47; 0.81)	0.68 (0.64; 0.72)
Ensemble models	8	0.78 (0.68; 0.85)	0.73 (0.58; 0.84)	0.83 (0.79; 0.86)
Deep learning models	1	0.61 (0.50; 0.71)	0.78 (0.62; 0.89)	0.70 (0.60; 0.80)

AI, artificial intelligence; AUC, area under the curve.

**Figure 3:**
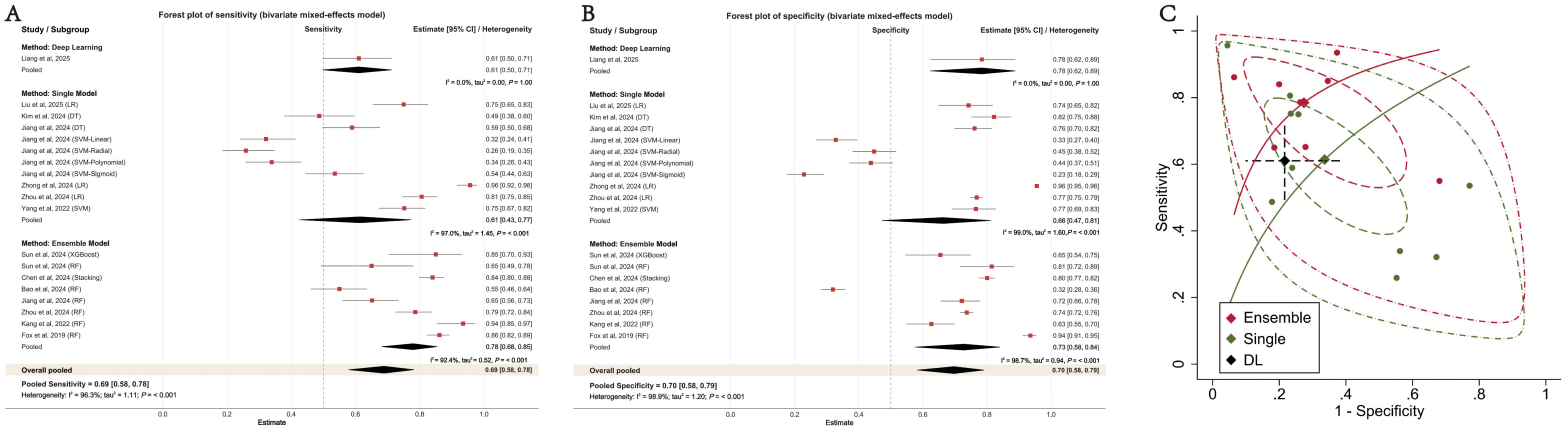
Forest and SROC plots of ML models for detecting NSSI. **(A)** Sensitivity **(B)** Specificity **(C)** SROC.

Only one study evaluated the performance of a DL model for NSSI, reporting an AUC of 0.70 (95% CI: 0.60–0.80), a sensitivity of 0.61 (95% CI: 0.50–0.71), and a specificity of 0.78 (95% CI: 0.62–0.89). This estimate should be interpreted cautiously. In the overall analysis of all 19 models, the pooled AUC was 0.75 (95% CI: 0.71–0.79), with a pooled sensitivity of 0.69 (95% CI: 0.58–0.78) and a pooled specificity of 0.70 (95% CI: 0.58–0.79). In the primary model-level analysis, ensemble models showed higher pooled discrimination than single models (Table 2 and Figure 3).

### 3.5 Heterogeneity across model-type subgroups

Substantial heterogeneity was observed both overall and within model-type subgroups. For sensitivity, the overall *I*^2^ was 96.3%, with subgroup *I*^2^ values of 97.0% for single models and 92.4% for ensemble models. For specificity, the overall *I*^2^ was 98.9%, with subgroup *I*^2^ values of 99.0% for single models and 98.7% for ensemble models. Heterogeneity could not be meaningfully assessed for the DL subgroup because it included only one study.

### 3.6 Sensitivity analysis

**Figure 4:**
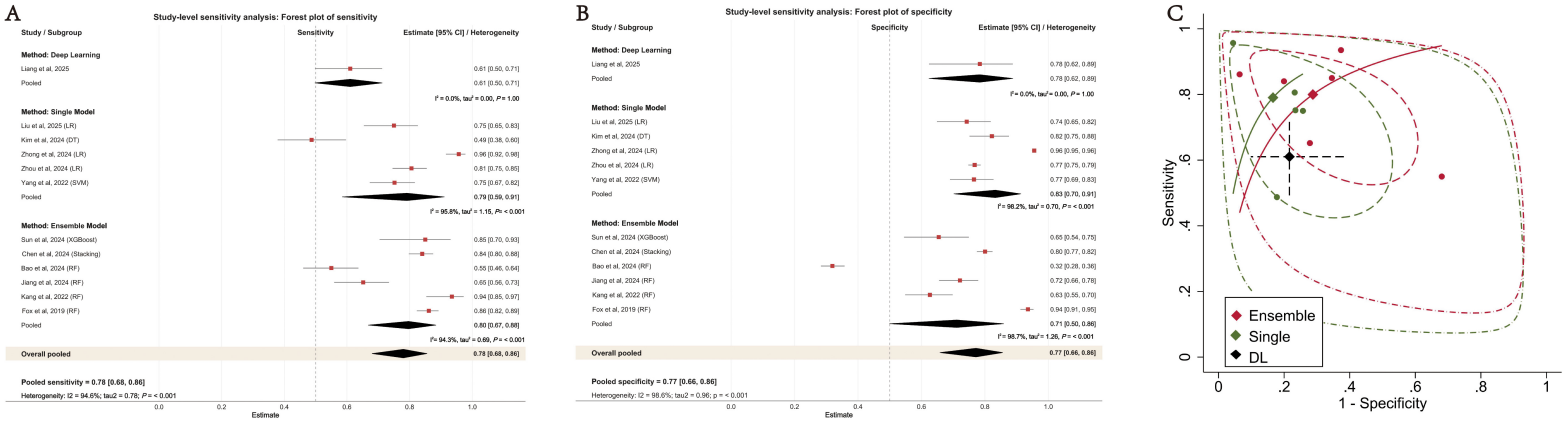
Forest and SROC plots of sensitivity analysis of ML models for detecting NSSI. **(A)** Sensitivity **(B)** Specificity **(C)** SROC.

In the study-level sensitivity analysis, one representative model from each study was retained, yielding 12 independent observations. Overall pooled performance remained favorable, with a sensitivity of 0.78 (95% CI: 0.68–0.86), a specificity of 0.77 (95% CI: 0.66–0.86), and an AUC of 0.85 (95% CI: 0.81–0.88). In subgroup analyses, single models showed a pooled sensitivity of 0.79 (95% CI: 0.59–0.91), a specificity of 0.83 (95% CI: 0.70–0.91), and an AUC of 0.89 (95% CI: 0.85–0.91), whereas ensemble models showed corresponding values of 0.80 (95% CI: 0.67–0.88), 0.71 (95% CI: 0.50–0.86), and 0.83 (95% CI: 0.80–0.86) (Table 3 and Figure 4). These findings differed from those of the primary model-level analysis, in which ensemble models showed higher pooled discrimination. After restricting the analysis to one representative model per study, this apparent advantage of ensemble models was no longer observed.

**Table 3:** Predictive performance of ML models for NSSI in the study-level sensitivity analysis, by model type.

AI algorithms	*n*	Sensitivity (95% CI)	Specificity (95% CI)	AUC (95% CI)
Single models	5	0.79 (0.59; 0.91)	0.83 (0.70; 0.91)	0.89 (0.85; 0.91)
Ensemble models	6	0.80 (0.67; 0.88)	0.71 (0.50; 0.86)	0.83 (0.80; 0.86)
Deep learning models	1	0.61 (0.50; 0.71)	0.78 (0.62; 0.89)	0.70 (0.60; 0.80)

AI, artificial intelligence; AUC, area under the curve.

### 3.7 Publication bias

**Figure 5:**
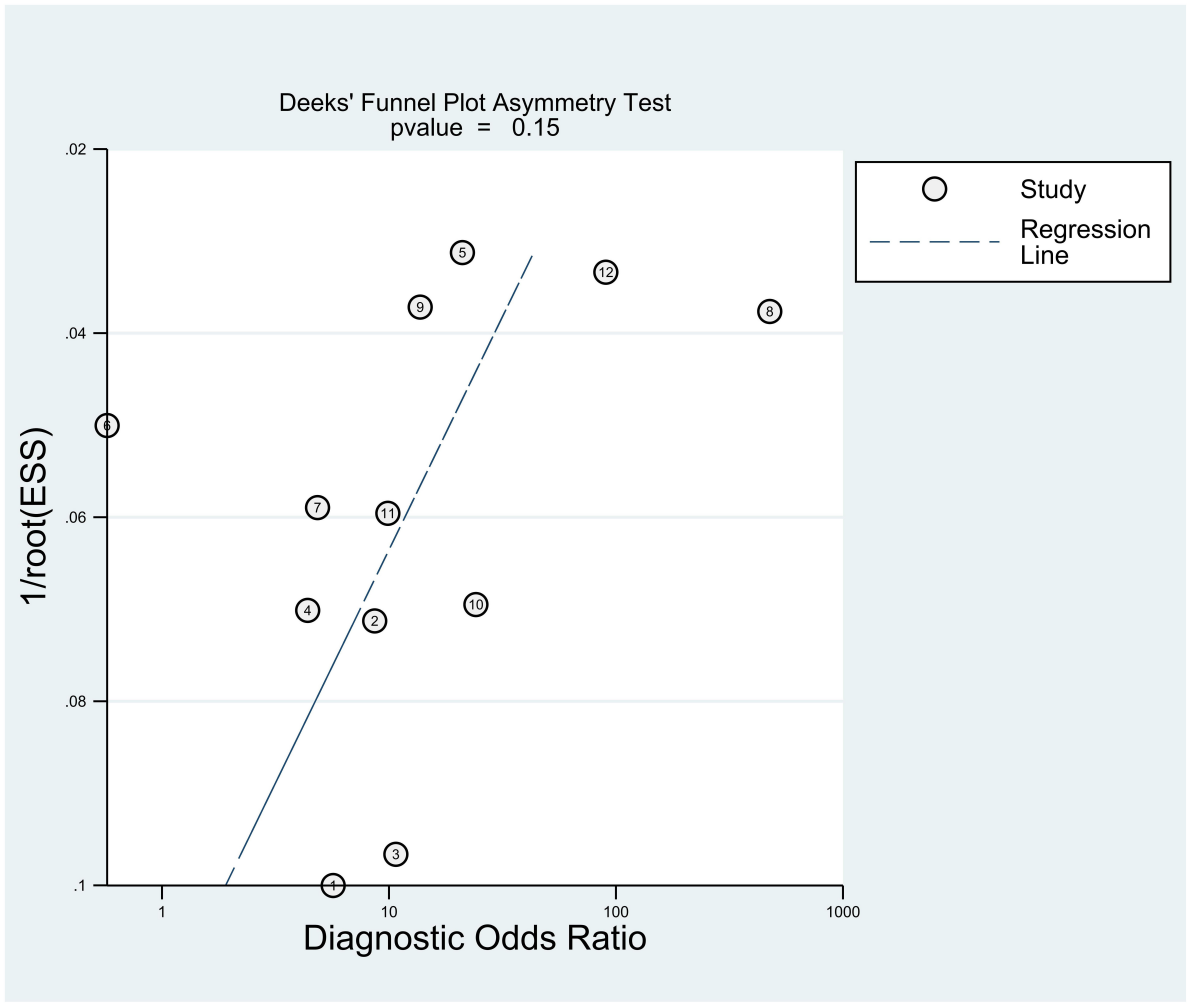
Deeks' funnel plot asymmetry test for publication bias.

Publication bias was assessed using Deeks' funnel plot asymmetry test and showed no statistically significant asymmetry (*t* = 1.57, *P* = 0.15) (Figure 5).

## 4 Discussion

This systematic review and meta-analysis synthesized the available evidence on ML-based prediction models for NSSI. Overall, ML models showed promising discriminative performance, with a pooled AUC of 0.75 across 19 models. In the primary model-level analysis, ensemble models appeared to outperform single models; however, this pattern was not retained in the study-level sensitivity analysis. Interpretation is further limited by substantial heterogeneity, frequent high risk of bias in the analysis domain, and the predominance of cross-sectional designs. Taken together, these findings suggest that ML may assist NSSI-related risk assessment, but current evidence remains insufficient to support routine clinical implementation.

A key strength of this review is its focus on NSSI as a distinct outcome. Although NSSI is associated with suicidal thoughts and behaviors, it is not interchangeable with suicidality and should not be treated simply as a proxy for suicide risk (36–38). This distinction matters clinically and methodologically, because it enables a more precise appraisal of model performance for NSSI itself.

Overall, the findings suggest that predictive signals for NSSI can be identified across a range of datasets and modeling approaches. This is noteworthy given the complex and multidetermined nature of NSSI, which arises from interacting emotional, psychiatric, developmental, interpersonal, and family-related factors and may, in some individuals, also involve neurobiological mechanisms (39–42). Conventional clinical assessment remains essential; however, its effectiveness is often constrained by its reliance on self-report, the difficulty of eliciting disclosure of self-harm and suicidal thoughts, and its limited capacity to integrate complex, high-dimensional risk information (43–45). In this context, ML may offer complementary value by integrating large, multidimensional risk indicators and identifying patterns that are difficult to detect using conventional approaches (46).

Findings related to model class warrant cautious interpretation. Although ensemble methods appeared to perform best in the primary model-level analysis, this apparent advantage was attenuated when the analysis was restricted to a single representative model from each study. This pattern suggests that the observed differences may partly reflect within-study model multiplicity rather than the robust and generalizable superiority of any particular algorithm family. In particular, these comparisons may be influenced by the inclusion of multiple correlated models derived from the same dataset, as well as by heterogeneity in sample characteristics, predictor selection, preprocessing procedures, outcome definitions, and validation strategies (47). Overall, the current evidence does not support definitive conclusions about the consistent superiority of any single ML approach for NSSI-related applications, particularly because the DL subgroup was represented by only one study.

The substantial heterogeneity observed in this review likely reflects both methodological diversity and clinical heterogeneity in NSSI itself (48). The included studies varied markedly in source populations, predictor domains, outcome definitions, reference periods, analytic workflows, and validation procedures. In parallel, NSSI is not a unitary phenotype: for some individuals it is more closely linked to affect regulation, whereas for others it may be more strongly associated with interpersonal stress or broader psychiatric burden (38). As a result, models developed in one setting may not generalize well to another. The pooled estimates should therefore be viewed as a summary of the current field rather than as universally transportable performance benchmarks.

An especially important issue is the distinction between concurrent identification and true prospective prediction. Several included studies were described as prediction studies, yet relied on cross-sectional or retrospective data and therefore more closely reflected classification of current or recent NSSI status than forecasting of future onset. This distinction is not semantic; it determines clinical utility. Models that identify individuals currently at elevated risk may still be useful for screening or triage, but they do not provide the same preventive value as models that can reliably predict future NSSI before onset (49). Accordingly, the present evidence supports NSSI risk identification more strongly than genuine longitudinal prediction.

The current findings nevertheless have practical implications. In schools, community mental health services, and outpatient settings, a model with moderate discriminative ability may still serve as a useful adjunct for screening, triage, and the prioritization of further psychosocial assessment, particularly when self-injury is concealed and help-seeking is inconsistent (50, 51). However, the available evidence does not support autonomous algorithm-based decision-making. At this stage, the most appropriate role of ML is to augment, rather than replace, clinical assessment within a broader framework of care.

This review also has several methodological strengths, including prospective registration, conduct and reporting in accordance with PRISMA 2020, and a structured risk-of-bias assessment using PROBAST+AI. In addition, when sufficient performance metrics were available, the reconstruction of 2 × 2 data enabled a more comparable quantitative synthesis across studies with heterogeneous reporting of model performance (52).

Several limitations should also be acknowledged. First, the evidence base remains methodologically immature. Most studies were judged to be at high risk of bias in the analysis domain, with recurring concerns including limited sample size, inadequate handling of missing data, overfitting, and insufficient validation. These issues are particularly important in ML research, where apparent performance may be overly optimistic when models are developed using relatively small datasets and evaluated solely through internal validation (53). Although Deeks' funnel plot asymmetry test did not indicate statistically significant asymmetry, this should not be interpreted as definitive evidence that publication bias or other small-study effects are absent, particularly given the modest number of studies and models and the substantial heterogeneity across analyses. Second, reporting was often incomplete. Key details regarding preprocessing, feature selection, threshold determination, and calibration were frequently missing, thereby limiting reproducibility and hindering comparisons across studies. Third, generalizability remains uncertain. Most included studies were conducted in China and relied primarily on structured questionnaire or clinical variables, whereas multimodal approaches involving EEG or neuroimaging were uncommon. Because sociocultural context may influence both the presentation of NSSI and the predictive value of specific features, the transportability of these models across settings cannot be assumed (54, 55).

The role of DL should likewise be interpreted with particular caution. Only one included study evaluated a DL approach; therefore, the available evidence is insufficient to determine whether DL offers any advantage or disadvantage in this field. Accordingly, no firm conclusion can be drawn regarding the relative performance of DL-based models for NSSI. At present, the central issue may be less whether DL is inherently superior than whether NSSI research has access to datasets that are sufficiently large, information-rich, and multimodal to support its appropriate application (56). Until such datasets become more widely available, conventional ML approaches may remain more practical, interpretable, and robust. However, greater attention to explainability is warranted in future NSSI prediction studies. Explainability analyses should be reported routinely alongside model performance metrics. For example, SHAP summary plots may help illustrate which predictors contribute most strongly to overall model output, whereas local SHAP visualizations may help explain individual high-risk predictions. Partial dependence plots may further clarify the direction and potential nonlinearity of predictor effects. Such reporting may improve transparency, facilitate clinical interpretation, and enhance clinical trust and translational relevance in model-assisted assessment (57).

Future research should prioritize methodological rigor, external validation, and clinical utility. Studies should adhere to contemporary guidance for model development and reporting, such as TRIPOD+AI and, where relevant, MI-CLAIM-GEN for generative AI applications, with more transparent reporting of calibration, threshold definition, and missing-data handling (19, 58). More robust validation strategies, including nested cross-validation for model tuning and evaluation in independent test sets, are needed to reduce optimism and provide more credible performance estimates (59). Larger, multicenter prospective cohorts with clearer outcome definitions are also required. Beyond discrimination, future studies should assess whether these models improve decision-making in real-world clinical or public health settings, including in distinguishing between NSSI alone and NSSI accompanied by elevated suicidal risk.

## 5 Conclusion

ML shows promise for improving the identification of NSSI risk, but the current evidence remains preliminary and methodologically limited. Although ensemble models achieved the highest pooled performance in the primary analysis, this apparent advantage was not consistent across sensitivity analyses and should not be interpreted as definitive evidence of algorithmic superiority. In particular, evidence regarding DL remains too limited to support any firm conclusion, as only one eligible study evaluated a DL model. Overall, the findings are encouraging but not yet sufficient to support routine clinical application. Future research should prioritize prospective study designs, external validation, transparent reporting, calibration assessment, and interpretability before ML models can be reliably translated into real-world NSSI risk assessment and prevention.

## Data Availability

The original contributions presented in the study are included in the article/Supplementary material, further inquiries can be directed to the corresponding author.

## Supplementary material

Supplementary Table S1 PRISMA (Preferred Reporting Items for Systematic Reviews and Meta-Analyses) checklist.

Supplementary Table S2 Literature search strategy.

Supplementary Table S3 Risk of bias assessment (PROBAST+AI) model development based on seven domains.

Supplementary Table S4 Risk of bias assessment (PROBAST+AI) model evaluation based on seven domains.
