# Supplementary material for: Can machine learning predict non-suicidal self-injury? A systematic review and meta-analysis
Source: Front Public Health. 2026 Apr 30;14:1763121. doi: 10.3389/fpubh.2026.1763121 (PMC13173908; doi:10.3389/fpubh.2026.1763121)
Supplement: Supplementary Table S2 — Literature search strategy. [file Supplementary_file_2.docx]

**Supplementary Table S2.** Literature search strategy

Search strategy (PubMed)

| **#** | **Query** | **Results** |
| --- | --- | --- |
| 1 | "Self-Injurious Behavior"[Mesh] | 90,842 |
| 2 | (((((((((((((((((((((((((Behavior, Self-Injurious[Title/Abstract]) OR (Self Injurious Behavior[Title/Abstract])) OR (Self-Injurious Behaviors[Title/Abstract])) OR (Intentional Self Injury[Title/Abstract])) OR (Intentional Self Injuries[Title/Abstract])) OR (Self Injury, Intentional[Title/Abstract])) OR (Intentional Self Harm[Title/Abstract])) OR (Self Harm, Intentional[Title/Abstract])) OR (Self-Destructive Behavior[Title/Abstract])) OR (Behavior, Self-Destructive[Title/Abstract])) OR (Self Destructive Behavior[Title/Abstract])) OR (Self-Destructive Behaviors[Title/Abstract])) OR (Deliberate Self-Harm[Title/Abstract])) OR (Deliberate Self Harm[Title/Abstract])) OR (Self-Harm, Deliberate[Title/Abstract])) OR (Self-Injury[Title/Abstract])) OR (Self Injury[Title/Abstract])) OR (Non-Suicidal Self Injury[Title/Abstract])) OR (Non-Suicidal Self Injuries[Title/Abstract])) OR (Non Suicidal Self Injury[Title/Abstract])) OR (Self Injury, Non-Suicidal[Title/Abstract])) OR (Self Harm[Title/Abstract])) OR (Harm, Self[Title/Abstract])) OR (Nonsuicidal Self Injury[Title/Abstract])) OR (Nonsuicidal Self Injuries[Title/Abstract])) OR (Self Injury, Nonsuicidal[Title/Abstract]) | 17,828 |
| 3 | #1 or #2 | 97,449 |
| 4 | "Machine Learning"[Mesh] | 95,978 |
| 5 | ((((((((((((((((((((((((((Learning, Machine[Title/Abstract]) OR (Transfer Learning[Title/Abstract])) OR (Learning, Transfer[Title/Abstract])) OR (Deep learning[Title/Abstract])) OR (Ensemble Learning[Title/Abstract])) OR (artificial intelligence[Title/Abstract])) OR (random forest[Title/Abstract])) OR (neural network[Title/Abstract])) OR (neural networks[Title/Abstract])) OR (K-Nearest Neighbor[Title/Abstract])) OR (CNN[Title/Abstract])) OR (Support vector machine[Title/Abstract])) OR (SVM[Title/Abstract])) OR (Gradient Boosting Machine[Title/Abstract])) OR (Nomogram[Title/Abstract])) OR (XGBoost[Title/Abstract])) OR (Adaboost[Title/Abstract])) OR (Decision tree[Title/Abstract])) OR (ResNet-50[Title/Abstract])) OR (ResNet[Title/Abstract])) OR (Naive Bayesian[Title/Abstract])) OR (Multilayer perceptron[Title/Abstract])) OR (Bayesian network[Title/Abstract])) OR (Radiomics[Title/Abstract])) OR (Radiomic[Title/Abstract])) OR (Prediction model[Title/Abstract])) OR (Risk model[Title/Abstract]) | 384,367 |
| 6 | #4 or #5 | 411,959 |
| 7 | #3 and #6 | 713 |

Search strategy (Web of Science)

| **#** | **Query** | **Results** |
| --- | --- | --- |
| 1 | (((((((((((((((((((((((((((TS=(Self-Injurious Behavior)) OR TS=(Behavior, Self-Injurious)) OR TS=(Self Injurious Behavior)) OR TS=(Self-Injurious Behaviors)) OR TS=(Intentional Self Injury)) OR TS=(Intentional Self Injuries)) OR TS=(Self Injury, Intentional)) OR TS=(Intentional Self Harm)) OR TS=(Self Harm, Intentional)) OR TS=(Self-Destructive Behavior)) OR TS=(Behavior, Self-Destructive)) OR TS=(Self Destructive Behavior)) OR TS=(Self-Destructive Behaviors)) OR TS=(Deliberate Self-Harm)) OR TS=(Deliberate Self Harm)) OR TS=(Self-Harm, Deliberate)) OR TS=(Self-Injury)) OR TS=(Self Injury)) OR TS=(Non-Suicidal Self Injury)) OR TS=(Non-Suicidal Self Injuries)) OR TS=(Non Suicidal Self Injury)) OR TS=(Self Injury, Non-Suicidal)) OR TS=(Self Harm)) OR TS=(Harm, Self)) OR TS=(Nonsuicidal Self Injury)) OR TS=(Nonsuicidal Self Injuries)) OR TS=(Self Injury, Nonsuicidal)) | 66,054 |
| 2 | (((((((((((((((((((((((((((TS=(Machine Learning)) OR TS=(Learning, Machine)) OR TS=(Transfer Learning)) OR TS=(Learning, Transfer)) OR TS=(Deep learning)) OR TS=(Ensemble Learning)) OR TS=(artificial intelligence)) OR TS=(random forest)) OR TS=(neural network)) OR TS=(neural networks)) OR TS=(K-Nearest Neighbor)) OR TS=(CNN)) OR TS=(Support vector machine)) OR TS=(SVM)) OR TS=(Gradient Boosting Machine)) OR TS=(Nomogram)) OR TS=(XGBoost)) OR TS=(Adaboost)) OR TS=(Decision tree)) OR TS=(ResNet-50)) OR TS=(ResNet)) OR TS=(Naive Bayesian)) OR TS=(Multilayer perceptron)) OR TS=(Bayesian network)) OR TS=(Radiomics)) OR TS=(Radiomic)) OR TS=(Prediction model)) OR TS=(Risk model) | 3,399,248 |
| 3 | #1 AND #2 | 7,252 |

Search strategy (Cochrane)

| **#** | **Query** | **Results** |
| --- | --- | --- |
| 1 | MeSH descriptor: [Self-Injurious Behavior] explode all trees | 2,675 |
| 2 | (Behavior, Self-Injurious)ti, ab, kw OR (Self Injurious Behavior)ti, ab, kw OR (Self-Injurious Behaviors)ti, ab, kw OR (Intentional Self Injury)ti, ab, kw OR (Intentional Self Injuries)ti, ab, kw OR (Self Injury, Intentional)ti, ab, kw OR (Intentional Self Harm)ti, ab, kw OR (Self Harm, Intentional)ti, ab, kw OR (Self-Destructive Behavior)ti, ab, kw OR (Behavior, Self-Destructive)ti, ab, kw OR (Self Destructive Behavior)ti, ab, kw OR (Self-Destructive Behaviors)ti, ab, kw OR (Deliberate Self-Harm)ti, ab, kw OR (Deliberate Self Harm)ti, ab, kw OR (Self-Harm, Deliberate)ti, ab, kw OR (Self-Injury)ti, ab, kw OR (Self Injury)ti, ab, kw OR (Non-Suicidal Self Injury)ti, ab, kw OR (Non-Suicidal Self Injuries)ti, ab, kw OR (Non Suicidal Self Injury)ti, ab, kw OR (Self Injury, Non-Suicidal)ti, ab, kw OR (Self Harm)ti, ab, kw OR (Harm, Self)ti, ab, kw OR (Nonsuicidal Self Injury)ti, ab, kw OR (Nonsuicidal Self Injuries)ti, ab, kw OR (Self Injury, Nonsuicidal)ti, ab, kw | 841 |
| 3 | #1 OR #2 | 3,502 |
| 4 | MeSH descriptor: [Machine Learning] explode all trees | 1,156 |
| 5 | (Learning, Machine)ti, ab, kw OR (Transfer Learning)ti, ab, kw OR (Learning, Transfer)ti, ab, kw OR (Deep learning)ti, ab, kw OR (Ensemble Learning)ti, ab, kw OR (artificial intelligence)ti, ab, kw OR (random forest)ti, ab, kw OR (neural network)ti, ab, kw OR (neural networks)ti, ab, kw OR (K-Nearest Neighbor)ti, ab, kw OR (CNN)ti, ab, kw OR (Support vector machine)ti, ab, kw OR (SVM)ti, ab, kw OR (Gradient Boosting Machine)ti, ab, kw OR (Nomogram)ti, ab, kw OR (XGBoost)ti, ab, kw OR (Adaboost)ti, ab, kw OR (Decision tree)ti, ab, kw OR (ResNet-50)ti, ab, kw OR (ResNet)ti, ab, kw OR (Naive Bayesian)ti, ab, kw OR (Multilayer perceptron)ti, ab, kw OR (Bayesian network)ti, ab, kw OR (Radiomics)ti, ab, kw OR (Radiomic)ti, ab, kw OR (Prediction model)ti, ab, kw OR (Risk model)ti, ab, kw | 2,278 |
| 6 | #4 OR #5 | 3,443 |
| 7 | #3 AND #6 | 832 |

Search strategy (Embase)

| **#** | **Query** | **Results** |
| --- | --- | --- |
| 1 | 'automutilation'/exp | 30,220 |
| 2 | 'Behavior, Self-Injurious':ab,ti OR 'Self Injurious Behavior':ab,ti OR 'Self-Injurious Behaviors':ab,ti OR 'Intentional Self Injury':ab,ti OR 'Intentional Self Injuries':ab,ti OR 'Self Injury, Intentional':ab,ti OR 'Intentional Self Harm':ab,ti OR 'Self Harm, Intentional':ab,ti OR 'Self-Destructive Behavior':ab,ti OR 'Behavior, Self-Destructive':ab,ti OR 'Self Destructive Behavior':ab,ti OR 'Self-Destructive Behaviors':ab,ti OR 'Deliberate Self-Harm':ab,ti OR 'Deliberate Self Harm':ab,ti OR 'Self-Harm, Deliberate':ab,ti OR 'Self-Injury':ab,ti OR 'Self Injury':ab,ti OR 'Non-Suicidal Self Injury':ab,ti OR 'Non-Suicidal Self Injuries':ab,ti OR 'Non Suicidal Self Injury':ab,ti OR 'Self Injury, Non-Suicidal':ab,ti OR 'Self Harm':ab,ti OR 'Harm, Self':ab,ti OR 'Nonsuicidal Self Injury':ab,ti OR 'Nonsuicidal Self Injuries':ab,ti OR 'Self Injury, Nonsuicidal':ab,ti | 20,670 |
| 3 | #1 OR #2 | 35,212 |
| 4 | 'machine learning'/exp | 602,596 |
| 5 | 'Learning, Machine':ab,ti OR 'Transfer Learning':ab,ti OR 'Learning, Transfer':ab,ti OR 'Deep learning':ab,ti OR 'Ensemble Learning':ab,ti OR 'artificial intelligence':ab,ti OR 'random forest':ab,ti OR 'neural network':ab,ti OR 'neural networks':ab,ti OR 'K-Nearest Neighbor':ab,ti OR 'CNN':ab,ti OR 'Support vector machine':ab,ti OR 'SVM':ab,ti OR 'Gradient Boosting Machine':ab,ti OR 'Nomogram':ab,ti OR 'XGBoost':ab,ti OR 'Adaboost':ab,ti OR 'Decision tree':ab,ti OR 'ResNet-50':ab,ti OR 'ResNet':ab,ti OR 'Naive Bayesian':ab,ti OR 'Multilayer perceptron':ab,ti OR 'Bayesian network':ab,ti OR 'Radiomics':ab,ti OR 'Radiomic':ab,ti OR 'Prediction model':ab,ti OR 'Risk model':ab,ti | 438,768 |
| 6 | #4 OR #5 | 790,885 |
| 7 | #3 AND #6 | 443 |

Search strategy ( PsycINFO)

| **S** | **Query** | **Results** |
| --- | --- | --- |
| 1 | XB “Self-Injurious Behavior” OR “Behavior, Self-Injurious” OR “Self Injurious Behavior” OR “Self-Injurious Behaviors” OR “Intentional Self Injury” OR “Intentional Self Injuries” OR “Self Injury, Intentional” OR “Intentional Self Harm” OR “Self Harm, Intentional” OR “Self-Destructive Behavior” OR “Behavior, Self-Destructive” OR “Self Destructive Behavior” OR “Self-Destructive Behaviors” OR “Deliberate Self-Harm” OR “Deliberate Self Harm” OR “Self-Harm, Deliberate” OR “Self-Injury” OR “Self Injury” OR “Non-Suicidal Self Injury” OR “Non-Suicidal Self Injuries” OR “Non Suicidal Self Injury” OR “Self Injury, Non-Suicidal” OR “Self Harm” OR “Harm, Self” OR “Nonsuicidal Self Injury” OR “Nonsuicidal Self Injuries” OR “Self Injury, Nonsuicidal” | 19,795 |
| 2 | XB “Machine Learning” OR “Learning, Machine” OR “Transfer Learning” OR “Learning, Transfer” OR “Deep learning” OR “Ensemble Learning” OR “artificial intelligence” OR “random forest” OR “neural network” OR “neural networks” OR “K-Nearest Neighbor” OR “CNN” OR “Support vector machine” OR “SVM” OR “Gradient Boosting Machine” OR “Nomogram” OR “XGBoost” OR “Adaboost” OR “Decision tree” OR “ResNet-50” OR “ResNet” OR “Naive Bayesian” OR “Multilayer perceptron” OR “Bayesian network” OR “Radiomics” OR “Radiomic” OR “Prediction model” OR “Risk model” | 108,149 |
| 3 | #1 AND #2 | 145 |

**STable6 : Search strategy ( IEEE Xplore).**

| **S** | **Query** | **Results** |
| --- | --- | --- |
| 1 | "All Metadata":"Self-Injurious Behavior" OR "All Metadata":"Behavior, Self-Injurious" OR "All Metadata":"Self Injurious Behavior" OR "All Metadata":"Self-Injurious Behaviors" OR "All Metadata":"Intentional Self Injury" OR "All Metadata":"Intentional Self Injuries" OR "All Metadata":"Self Injury, Intentional" OR "All Metadata":"Intentional Self Harm" OR "All Metadata":"Self Harm, Intentional" OR "All Metadata":"Self-Destructive Behavior" OR "All Metadata":"Behavior, Self-Destructive" OR "All Metadata":"Self Destructive Behavior" OR "All Metadata":"Self-Destructive Behaviors" OR "All Metadata":"Deliberate Self-Harm" OR "All Metadata":"Deliberate Self Harm" OR "All Metadata":"Self-Harm, Deliberate" OR "All Metadata":"Self-Injury" OR "All Metadata":"Self Injury" OR "All Metadata":"Non-Suicidal Self Injury" OR "All Metadata":"Non-Suicidal Self Injuries" OR "All Metadata":"Non Suicidal Self Injury" OR "All Metadata":"Self Injury, Non-Suicidal" OR "All Metadata":"Self Harm" OR "All Metadata":"Harm, Self" OR "All Metadata":"Nonsuicidal Self Injury" OR "All Metadata":"Nonsuicidal Self Injuries" OR "All Metadata":"Self Injury, Nonsuicidal" | 106 |
| 2 | "All Metadata":"Machine Learning" OR "All Metadata":"Learning, Machine" OR "All Metadata":"Transfer Learning" OR "All Metadata":"Learning, Transfer" OR "All Metadata":"Deep learning" OR "All Metadata":"Ensemble Learning" OR "All Metadata":"artificial intelligence" OR "All Metadata":"random forest" OR "All Metadata":"neural network" OR "All Metadata":"neural networks" OR "All Metadata":"K-Nearest Neighbor" OR "All Metadata":"CNN" OR "All Metadata":"Support vector machine" OR "All Metadata":"SVM" OR "All Metadata":"Gradient Boosting Machine" OR "All Metadata":"Nomogram" OR "All Metadata":"XGBoost" OR "All Metadata":"Adaboost" OR "All Metadata":"Decision tree" OR "All Metadata":"ResNet-50" OR "All Metadata":"ResNet" OR "All Metadata":"Naive Bayesian" OR "All Metadata":"Multilayer perceptron" OR "All Metadata":"Bayesian network" OR "All Metadata":"Radiomics" OR "All Metadata":"Radiomic" OR "All Metadata":"Prediction model" OR "All Metadata":"Risk model" | 1,087,368 |
| 3 | #1 AND #2 | 68 |
