# Supplementary material for: Can machine learning predict non-suicidal self-injury? A systematic review and meta-analysis
Source: Front Public Health. 2026 Apr 30;14:1763121. doi: 10.3389/fpubh.2026.1763121 (PMC13173908; doi:10.3389/fpubh.2026.1763121)
Supplement: Supplementary Table S3 — Risk of bias assessment (PROBAST+AI) model development based on seven domains. [file Supplementary_file_3.docx]

**Supplementary Table S3**. Risk of bias assessment (PROBAST+AI) model development based on seven domains.

| Author, year | | Quality | | | | Applicability concerns | | | Overall judgement | |
| --- | --- | --- | --- | --- | --- | --- | --- | --- | --- | --- |
|  |  | Participants and data sources ^a^ | Predictors ^b^ | Outcome ^c^ | Analysis ^d^ | Participants and data sources ^e^ | Predictors ^f^ | Outcome ^g^ | Quality ^h^ | Applicability concerns ^i^ |
| Liang et al. | 2025 | L | L | L | L | L | L | L | L | L |
| Liu et al. | 2025 | L | L | U | L | L | L | L | U | L |
| Sun et al. | 2024 | L | U | U | U | L | L | L | U | L |
| Kim et al. | 2024 | L | L | L | U | L | L | L | U | L |
| Chen et al. | 2024 | L | L | L | L | L | L | L | L | L |
| Bao et al. | 2024 | H | L | U | L | L | L | L | H | L |
| Jiang et al. | 2024 | L | L | L | L | L | L | L | L | L |
| Zhong et al. | 2024 | L | L | L | U | L | L | L | U | L |
| Zhou et al. | 2024 | L | L | L | U | L | L | L | U | L |
| Kang et al. | 2022 | H | U | U | U | L | L | L | H | L |
| Yang et al. | 2022 | H | L | L | U | L | L | L | H | L |
| Fox et al. | 2019 | L | L | L | L | L | L | L | L | L |

**Abbreviation:** PROBAST+AI, Prediction model Risk of Bias Assessment Tool + AI, L low; H high; U unclear.

**Footnote:** Signaling questions are rated as "yes" (Y), "probably yes" (PY), "probably no" (PN), "no" (N), "no information" (NI), and in some cases "not applicable" (NA). All signaling questions are phrased in such a way that "yes" or "probably yes" indicates a low risk of bias. Any signaling questions rated as "no" or "probably no" indicate a potential high risk of bias in that domain. If there are no "no" or "probably no" ratings, but "no information" (NI) is present, the risk of bias in that domain is classified as unclear.

**a. Participants and data sources**

1.1 Were appropriate data sources used?

1.2 Was an appropriate study design used?

1.3 Did the in- and exclusions of study participants result in a representative dataset?

**b. Predictors**

2.1 Were predictors defined and assessed in a similar way for all participants?

2.2 Was any pre-processing of predictors similar for all participants?

2.3 Were predictor assessments made without knowledge of outcome data?

2.4 Were the predictors included in the model available at the time the model was intended to be used?

**c. Outcome**

3.1 Were outcomes defined and assessed appropriately?

3.2 Were outcomes defined and assessed in a similar way for all participants?

3.3 Were outcome assessments made without use or knowledge of predictor data?

3.4 Was the time interval between predictor assessment and outcome assessment appropriate?

**d. Analysis**

4.1 Was there evidence that the sample size was reasonable?

4.2 Were continuous and categorical predictors handled appropriately?

4.3 Were participants with missing or censored data handled appropriately in the analysis?

4.4 If methods to address class imbalance were used, was the model or the model predictions recalibrated?

4.5 Were methods used to address potential model overfitting?

**e. Participants and data sources**

Concern that the (data of the) included participants do not match the review question or the assessor’s intended use of the prediction model.

f. **Predictors**

Concern that the definition, pre-processing, assessment, or timing of assessment of the predictors in the model do not match the review question or the assessor’s intended use.

g. **Outcome**

Concern that the outcome, its definition, assessment, or timing of assessment do not match the review question or the assessor’s intended use.

**h. Quality**

Low risk: If all four domains were rated low concern regarding quality.

High risk: If at least one domain was rated high concern regarding quality .

Unclear: If at least one domain was rated unclear concern regarding quality and no domains were rated high concern.

**i. Applicability concerns**

Low risk: If all three domains were rated low concern for applicability.

High risk: If at least one domain was rated high concern for applicability.

Unclear: If at least one domain was rated unclear concern for applicability and no domains were rated high concern.
